# The impact of surface Cu^2+^ of ZnO/(Cu_1−*x*_Zn_*x*_)O heterostructured nanowires on the adsorption and chemical transformation of carbonyl compounds†

**DOI:** 10.1039/d1sc00729g

**Published:** 2021-03-08

**Authors:** Jiangyang Liu, Kazuki Nagashima, Yuki Nagamatsu, Takuro Hosomi, Hikaru Saito, Chen Wang, Wataru Mizukami, Guozhu Zhang, Benjarong Samransuksamer, Tsunaki Takahashi, Masaki Kanai, Takao Yasui, Yoshinobu Baba, Takeshi Yanagida

**Affiliations:** Department of Applied Chemistry, Graduate School of Engineering, The University of Tokyo 7-3-1 Hongo Bunkyo-ku Tokyo 113-8656 Japan kazu-n@g.ecc.u-tokyo.ac.jp yanagida@g.ecc.u-tokyo.ac.jp; Japan Science and Technology Agency (JST), PRESTO 4-1-8 Honcho, Kawaguchi Saitama 332-0012 Japan; Institute for Materials Chemistry and Engineering, Kyushu University 6-1 Kasuga-Koen Kasuga Fukuoka 816-8580 Japan; Center for Quantum Information and Quantum Biology, Institute for Open and Transdisciplinary Research Initiatives, Osaka University 1-3 Machikaneyama Toyonaka Osaka 560-8531 Japan; Graduate School of Engineering Science, Osaka University 1-3 Machikaneyama Toyonaka Osaka 560-8531 Japan; Department of Biomolecular Engieering, Graduate School of Engineering, Nagoya University Furo-cho, Chikusa-ku Nagoya 464-8603 Japan

## Abstract

The surface cation composition of nanoscale metal oxides critically determines the properties of various functional chemical processes including inhomogeneous catalysts and molecular sensors. Here we employ a gradual modulation of cation composition on a ZnO/(Cu_1−*x*_Zn_*x*_)O heterostructured nanowire surface to study the effect of surface cation composition (Cu/Zn) on the adsorption and chemical transformation behaviors of volatile carbonyl compounds (nonanal: biomarker). Controlling cation diffusion at the ZnO(core)/CuO(shell) nanowire interface allows us to continuously manipulate the surface Cu/Zn ratio of ZnO/(Cu_1−*x*_Zn_*x*_)O heterostructured nanowires, while keeping the nanowire morphology. We found that surface exposed copper significantly suppresses the adsorption of nonanal, which is not consistent with our initial expectation since the Lewis acidity of Cu^2+^ is strong enough and comparable to that of Zn^2+^. In addition, an increase of the Cu/Zn ratio on the nanowire surface suppresses the aldol condensation reaction of nonanal. Surface spectroscopic analysis and theoretical simulations reveal that the nonanal molecules adsorbed at surface Cu^2+^ sites are not activated, and a coordination-saturated in-plane square geometry of surface Cu^2+^ is responsible for the observed weak molecular adsorption behaviors. This inactive surface Cu^2+^ well explains the mechanism of suppressed surface aldol condensation reactions by preventing the neighboring of activated nonanal molecules. We apply this tailored cation composition surface for electrical molecular sensing of nonanal and successfully demonstrate the improvements of durability and recovery time as a consequence of controlled surface molecular behaviors.

## Abstract 2

Unexpected features of surface Cu^2+^ on ZnO/(Cu1−*x*Zn*x*)O nanowires for molecular transformation and electrical sensing of carbonyl compounds were found.

## Graphical abstract

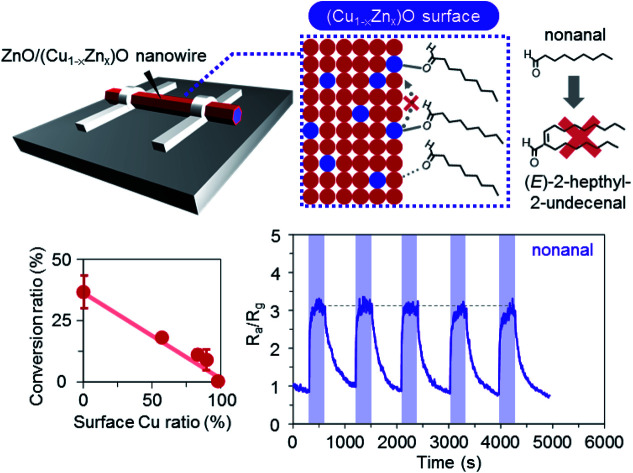

## Introduction

The surface cation composition of nanoscale metal oxides is well known to significantly affect the surface molecular behaviors in various applications including inhomogeneous catalysts, molecular sensing, and others.^1–5^ This is because various molecular reaction pathways are induced *via* interacting sequentially or simultaneously with different cation species on the surface.^6–9^ Thus, investigating the effect of surface cation composition on the surface molecular behaviors is of crucial importance for designing the properties of the above applications. However, it remains a challenging issue to alter systematically and precisely the surface cation composition of nanoscale metal oxides without changing the macroscopic morphology.^10,11^ Cation exchange and cation diffusion are promising ways to tailor the surface cation composition of nanoscale metal oxides.^12–17^ For example, the surface cation of Fe_3_O_4_ nanoparticles was substituted with Co and Mn *via* cation exchange in a wet process^12^ and ZnO/ZnCo_2_O_4_ core/shell nanowires were synthesized from ZnO nanobelts *via* cation exchange in a dry process.^13,14^ Cation diffusion offers a more facile and versatile approach for controlling the composition of nanoscale metal oxides; *e.g.* Mn-doped surface of TiO_2_ nanowires and ZnO/In_*x*_Zn_1−*x*_O axial heteronanowires were successfully demonstrated with a controllable surface cation composition.^15–17^ As such these approaches allow us to widely and systematically control the surface cation composition of nanoscale metal oxides while keeping their macroscopic morphology.

Here we demonstrate a gradual modulation of the surface cation composition of heterostructured metal oxide nanowires *via* cation diffusion. A ZnO/(Cu_1−*x*_Zn_*x*_)O heterostructured nanowire is employed as a model. We found that the surface Cu/Zu ratio of the ZnO/(Cu_1−*x*_Zn_*x*_)O heterostructured nanowire is widely modulated and the effect of the surface Cu/Zn ratio on the molecular transformation behavior of volatile carbonyl compounds (nonanal: biomarker) is systematically investigated. CuO, ZnO and their composite are abundant and often utilized for molecular sensing and oxidizing/cyclotrimerizing catalyst of carbonyl compounds.^18–22^ Carbonyl compounds are important as initiators for organic synthesis^23,24^ and also known as target molecules for environmental, industrial and biomedical sensing.^25–27^ By continuously modulating the surface Cu/Zn ratio of the ZnO/(Cu_1−*x*_Zn_*x*_)O heterostructured nanowires, we found the unexpected properties of surface Cu^2+^ on the adsorption of nonanal. Surface spectroscopic analysis and theoretical simulations provide the mechanistic understanding of the role of surface Cu^2+^. Furthermore, we demonstrate an application of the tailored surface cation composition of ZnO/(Cu_1−*x*_Zn_*x*_)O heterostructured nanowires for improving the performance of electrical molecular sensing of nonanal.

## Results and discussion

**Fig. 1:**
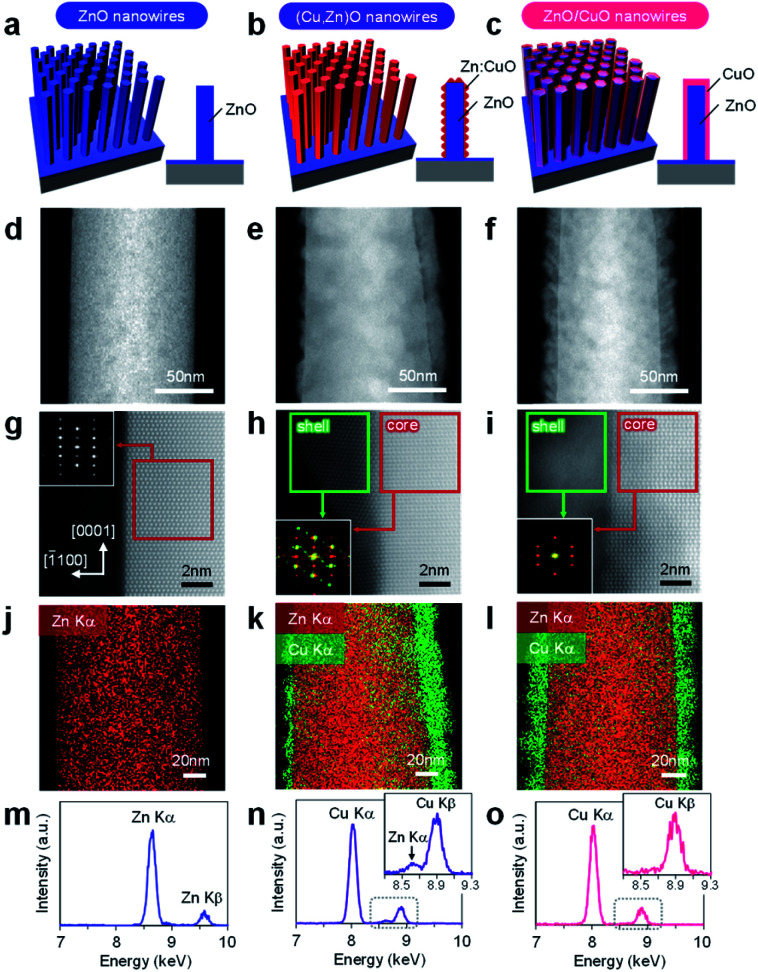
(a–c) Schematic illustrations, (d–f) low magnification and (g–i) high magnification ADF-STEM images, (j–l) EDS elemental maps and (m–o) EDS spectra of (a, d, g, j, and m) ZnO nanowires, (b, e, h, k, and n) (Cu, Zn)O nanowires and (c, f, i, l, and o) ZnO/CuO nanowires, respectively. The insets of (g–i) show FFT patterns. The employed CuO shell thickness is *ca.* 10 nm.

**Fig. 2:**
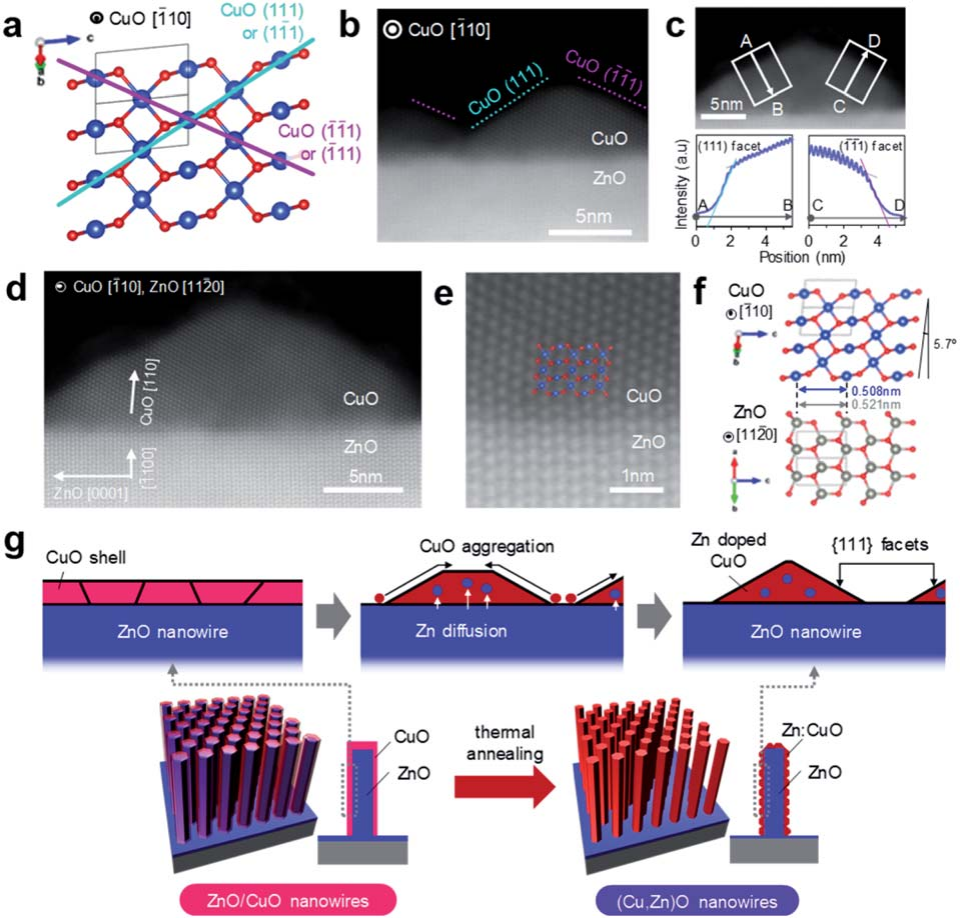
(a) Crystal structure of monoclinic CuO. (b) ADF-STEM image of the (Cu, Zn)O nanowire surface. (c) ADF-STEM image of the CuO shell (upper) and image intensity profiles along the A–B and C–D lines perpendicular to the CuO (111) and CuO (1̄1̄1) facets (lower). (d) TEM image and (e) high-resolution TEM image of the (Cu, Zn)O nanowire at the interface. (f) Crystal structures of ZnO and CuO and their orientations at the interface. (g) Schematic illustration for the formation process of the (Cu, Zn)O nanowire surface *via* cation diffusion.

In this study, cation diffusion is employed to gradually modulate the surface cation composition of core/shell heterostructured nanowires. Fig. 1a–c show the schematic images for three types of nanowire samples utilized in this study: (i) ZnO nanowires, (ii) (Cu, Zn)O nanowires and (iii) ZnO/CuO nanowires, where (Cu, Zn)O nanowires and ZnO/CuO nanowires represent CuO shell coated ZnO nanowires with and without thermal annealing, respectively (see details in the ESI, Fig. S1–S4 and Table S1).† A heterocompositional surface of ZnO/(Cu_1−*x*_Zn_*x*_)O nanowires is formed by the cation diffusion at the ZnO(core)/CuO(shell) interface during thermal annealing^28–30^ (see details in the ESI, Fig. S1–S4 and Table S1†). We varied the CuO shell thickness and the annealing temperature as parameters to control the surface cation composition, while keeping the annealing time of 3 h. The results of annealing temperature dependence (ESI Fig. S1†) show that 400–500 °C is the appropriate temperature range for cation diffusion by preserving the nanowire morphology. Fig. 1d–f show the annular dark field-scanning transmission electron microscopy (ADF-STEM) images of the ZnO nanowire, (Cu, Zn)O nanowire (annealed at 400 °C) and ZnO/CuO nanowire, respectively. The CuO shell thickness is *ca.* 10 nm. While the CuO shell layer fully covers the nanowire surface in the ZnO/CuO nanowire, the ZnO nanowire surface is partly exposed after thermal annealing (*i.e.* (Cu, Zn)O nanowire). High magnification ADF-STEM images and fast Fourier transform (FFT) patterns in Fig. 1g–i indicate that the ZnO core nanowire is single crystalline and the CuO shell layer changes from an amorphous to a polycrystalline structure by thermal annealing. The ZnO nanowire exposes {101̄0} facets on the surface. The energy dispersive X-ray spectroscopy (EDS) based elemental maps in Fig. 1j–l and ESI S5–S7† also support the observed morphological change of the (Cu, Zn)O nanowire surface. The EDS spectra obtained at the nanowire surface (Fig. 1m–o and ESI S5–S7†) show the occurrence of Zn diffusion into the CuO shell layer during thermal annealing.^28–30^ The distribution of Zn (*ca.* 4.5%) is homogeneous over the CuO shell layer (ESI Fig. S7†). Note that Cu diffusion into the ZnO nanowire is not identified (ESI Fig. S8†). The observed all structural and compositional characteristics are also seen in the (Cu, Zn)O nanowire samples annealed at 500 °C, while the Zn concentration in CuO is *ca.* 2.3%. We found that CuO forms an epitaxial interface at CuO (110)/ZnO (101̄0) with a crystal orientation relationship [1̄10] CuO ‖ [112̄0] ZnO and exposes {111} facets at the surface during the crystallization process as shown in Fig. 2a–f and ESI S9.† To the best of our knowledge, the epitaxial interface of CuO (110)/ZnO(101̄0) is reported in this study for the first time. The steep intensity profiles across the CuO surface confirm the presence of {111} facets (Fig. 2c). The formation of {111} facets on the CuO shell surface is consistent with the fact that {111} planes have the lowest surface energy.^31,32^ Thus these results show that the heterocompositional surface of Zn-doped CuO and partly exposed ZnO is formed on the ZnO/(Cu_1−*x*_Zn_*x*_)O nanowire by cation diffusion (as schematically shown in Fig. 2g).

**Fig. 3:**
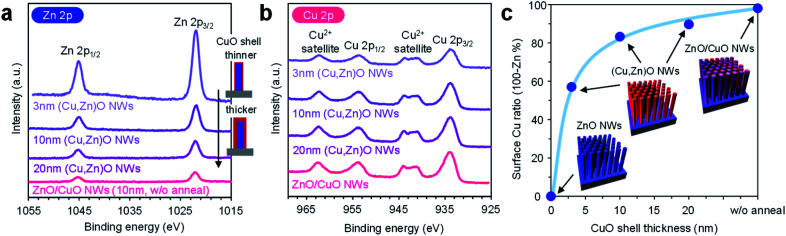
(a) Zn 2p and (b) Cu 2p XPS spectra of ZnO/CuO nanowires and (Cu, Zn)O nanowires. (c) Surface Cu ratio of the nanowires as a function of CuO shell thickness. For ZnO/CuO nanowires, the CuO shell thickness is 10 nm and oxygen plasma treatment is performed prior to measurement. For (Cu, Zn)O nanowires, the CuO shell thickness is varied in the range of 3–20 nm. ‘NWs’ represents ‘nanowires’.

In order to characterize the cation composition of nanowire surfaces, X-ray photoemission spectroscopy (XPS) was performed. Fig. 3a and b show the Zn 2p and Cu 2p XPS spectra of ZnO/CuO nanowires (CuO layer: 10 nm thickness) and (Cu, Zn)O nanowires (CuO layer: 3–20 nm thickness, annealed at 500 °C). For the ZnO/CuO nanowires, oxygen plasma treatment is applied prior to the measurement in order to control the valence state of the CuO shell to be comparable with that of (Cu, Zn)O nanowires (ESI Fig. S10†). We found that the Zn 2p peak of ZnO/CuO nanowires is the smallest, and those of (Cu, Zn)O nanowires tend to be larger with decreasing the CuO shell thickness. Note that the intensity of Cu 2p peaks and the major valence state of Cu^2+^ are similar among these samples. Fig. 3c shows the surface Cu ratio of the nanowires as a function of CuO shell thickness. In this figure, the data of ZnO/CuO nanowires with a 10 nm CuO shell are also shown. The surface Cu ratio of the nanowires decreases from 98% (in ZnO/CuO nanowires) to 83% (in (Cu, Zn)O nanowires) by thermal annealing. Note that a small percent of zinc detected in ZnO/CuO nanowires is plausibly due to the imperfect coating of the CuO layer at the bottom part of the ZnO nanowires. The surface Cu ratio of the nanowires is widely and gradually modulated from 0% to 98% among the examined samples (ZnO nanowires, ZnO/CuO nanowires and (Cu, Zn)O nanowires with varying the CuO shell thickness). By taking into account the fact that the distribution of Zn in the CuO shell is homogeneous, the decrease of the surface Cu ratio when decreasing the CuO shell thickness is mainly caused by the increase of the exposed area of ZnO core nanowires. The results highlight that the examined post-growth annealing treatment allows us to gradually and widely modulate the surface Cu/Zn ratio of the nanowires by preserving the macroscopic morphology.

**Fig. 4:**
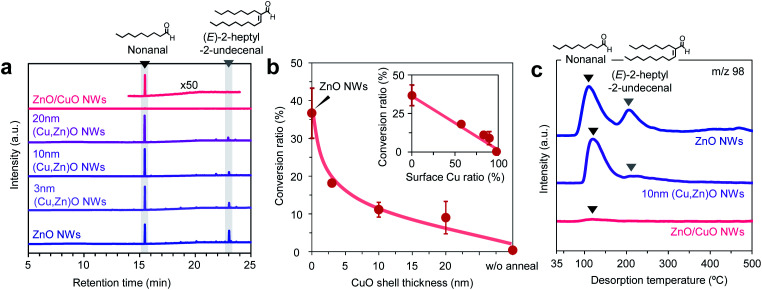
(a) GCMS spectra for desorbed compounds on ZnO nanowires, ZnO/CuO nanowires and (Cu, Zn)O nanowires. (b) CuO shell thickness dependent conversion ratio of nonanal to *(E)*-2-hepthyl-2-undecenal. The inset shows its surface Cu ratio dependence. (c) TPDMS profiles of desorbed compounds on ZnO nanowires, ZnO/CuO nanowires and (Cu, Zn)O nanowires, which are evaluated at *m*/*z* 98. For ZnO/CuO nanowires, the CuO shell thickness is 10 nm. For (Cu, Zn)O nanowires, the CuO shell thickness is varied in the range of 3–20 nm. ‘NWs’ represents ‘nanowires’.

Next we investigate the effect of the Cu/Zn ratio of nanowires on the adsorption and the chemical transformation of volatile carbonyl compound molecules. Fig. 4a shows the gas chromatography mass spectrometry (GCMS) spectra of desorbed compounds from the nonanal-adsorbed ZnO nanowires, ZnO/CuO nanowires and (Cu, Zn)O nanowires (annealed at 500 °C). The 50 times amplified spectrum for ZnO/CuO nanowires is also shown to highlight the presence of the peak. Consistent with our previous report, two distinct peaks assigned to nonanal and its aldol condensation product, *(E)*-2-heptyl-2-undecenal, are seen in the spectrum of ZnO nanowires.^33^ On the other hand, only a small nonanal peak is observed in ZnO/CuO nanowires, implying that both the adsorption and the surface reaction of nonanal are suppressed on the CuO surface. These are unexpected results because the Lewis acidity of Cu^2+^ is strong enough and comparable to that of Zn^2+^.^34^ For the (Cu, Zn)O nanowires, the *(E)*-2-heptyl-2-undecenal peak is smaller than that for the ZnO nanowires. Also the intensity of the *(E)*-2-heptyl-2-undecenal peak tends to be smaller with maintaining the nonanal peak when increasing the CuO shell thickness. Fig. 4b shows the conversion ratio of nonanal to *(E)*-2-hepthyl-2-undecenal on the nanowires. The conversion ratio of nonanal is estimated *via* the amount ratio of desorbed compounds. The conversion ratio decreases from 36.7% to 9.0% when varying the CuO shell thickness from 0 nm to 20 nm in (Cu, Zn)O nanowires. An inverse linear relationship between the conversion ratio and the surface Cu ratio (inset of Fig. 4b) clearly indicates that the surface condensation reaction is suppressed by the surface exposed copper. This also indicates that a synergistic effect between Cu and Zn seems to be not significant for the conversion efficiency. Fig. 4c shows the temperature-programmed desorption mass spectrometry (TPDMS) profiles of desorption compounds from the nonanal-adsorbed nanowires. Two peaks associated with nonanal (peak top is *ca.* 115 °C) and *(E)*-2-hepthyl-2-undecenal (peak top is *ca.* 230 °C) are seen in the spectrum of ZnO nanowires while only a small nonanal peak is seen in the ZnO/CuO nanowires. The *(E)*-2-hepthyl-2-undecenal peak is drastically suppressed in (Cu, Zn)O nanowires, while maintaining the desorption temperature of nonanal. These results highlight the significant effect of surface Cu^2+^ on the surface molecular behaviors of nonanal and also suggest that the surface condensation reaction can be tailored while maintaining the adsorption by controlling the surface Cu/Zn ratio on (Cu, Zn)O nanowires.

**Fig. 5:**
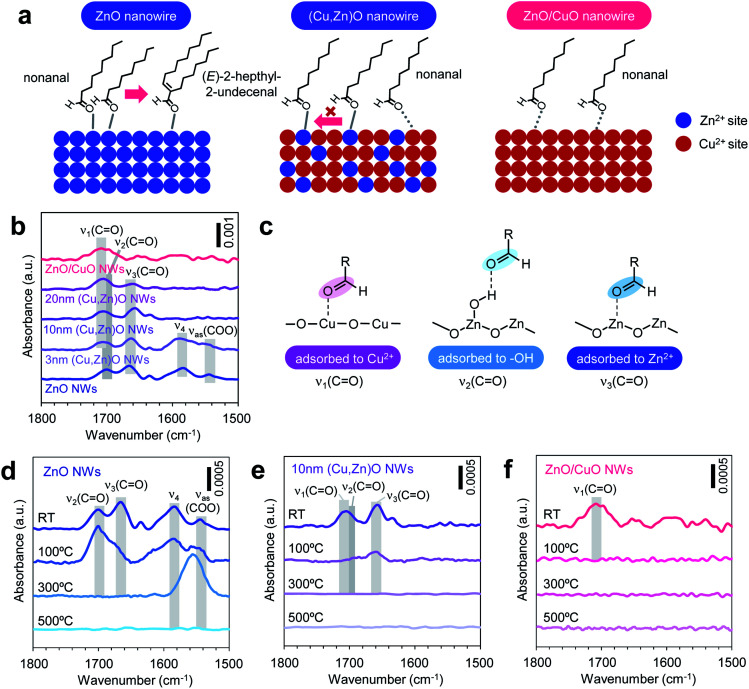
(a) Schematic illustrations for nonanal adsorption and aldol condensation events on the ZnO nanowire surface (left), ZnO/CuO nanowire surface (middle) and (Cu, Zn)O nanowire surface (right). (b) FTIR spectra of nonanal-adsorbed ZnO nanowires, ZnO/CuO nanowires and (Cu, Zn)O nanowires. (c) Adsorption structure of C=O at the Cu^2+^ site (*ν*_1_), –OH site (*ν*_2_) and Zn^2+^ site (*ν*_3_). (d–f) Temperature dependent variations of FTIR spectra for nonanal adsorbed on (d) ZnO nanowires, (e) (Cu, Zn)O nanowires and (f) ZnO/CuO nanowires after baking at a given temperature for 5 min. For all FTIR spectra, background signals from the nanowires are subtracted. For ZnO/CuO nanowires, the CuO shell thickness is 10 nm. For (Cu, Zn)O nanowires, the CuO shell thickness is varied in the range of 3–20 nm. ‘NWs’ and ‘RT’ represent ‘nanowires’ and ‘room temperature’, respectively.

Here we question why the surface Cu^2+^ significantly suppressed the adsorption and the chemical transformation of nonanal while the Lewis acidities of Cu^2+^ and Zn^2+^ are comparable.^34^ We consider the effect of the coordination degree of exposed surface metal ions, which influences the adsorption of molecules. The coordination degree of surface metal ions is inherently complex, but generally depends on the crystal structure and crystal plane.^35^ On the CuO surface, the CuO (111) plane, which has the lowest surface energy, is likely to be exposed *via* surface reconstruction during thermal annealing (Fig. 2) and oxygen plasma treatment.^31,32^ Since the Cu^2+^ ion on the CuO (111) plane has a coordination-saturated in-plane square geometry, the molecular adsorption on the Cu^2+^ site must be destabilized compared with that on the Zn^2+^ ion of the ZnO (101̄0) plane (*i.e.* sidewall plane), which has a coordination-unsaturated tetrahedral geometry. In fact, our density functional theory (DFT) calculations show that the adsorption energy of C=O − M^+^ (*i.e.* nonanal-metal ion bonding) becomes much lower on the Cu^2+^ site than that on the Zn^2+^ site by introducing the coordination geometry of metal ions (ESI Fig. S11–S12 and Table S2†). This calculation result is also supported by the molecular adsorption analyses on CuO thin films without ZnO nanowires, in which quite a limited amount of nonanal is identified in their desorbed compounds (ESI Fig. S13†). Thus, the observed weak adsorption behaviors of nonanal on the CuO surface in Fig. 4a can be interpreted in terms of the coordination-saturated geometry of the Cu^2+^ ion. The above analogy is also applicable to explain why the surface aldol condensation reaction is suppressed on the (Cu, Zn)O nanowire surface. The observed molecular adsorption behaviors suggest that nonanal molecules are not activated at the Cu^2+^ site of (Cu, Zn)O nanowires. Since our previous study revealed that the aldol condensation reaction is triggered by the neighboring of two nonanal molecules activated at adjacent Zn^2+^ Lewis acidic sites on ZnO nanowires (Fig. 5a, left),^33^ such a reaction can be suppressed by preventing the neighboring of Zn^2+^ sites on the nanowire surface (Fig. 5a, middle). The prevention of neighboring of Zn^2+^ sites is expected on the Zn-doped CuO surface due to its low Zn concentration (*ca.*2.3–4.5%). On the ZnO-exposed area, the aldol condensation reaction would occur, yet its probability must be drastically lowered at the periphery of the ZnO/CuO interface due to the absence of neighbored Zn^2+^ sites (ESI Fig. S14†). The effects of the inactive Cu^2+^ site and ZnO/CuO interfacial periphery become more significant when increasing the surface Cu/Zn ratio. Thus the aldol condensation reaction is suppressed with increasing the surface Cu/Zn ratio. Note that the surface molecular behaviors on (Cu, Zn)O nanowires and ZnO/CuO nanowires must be different; nonanal mainly adsorbs at the isolated Zn^2+^ site on the (Cu, Zn)O nanowire surface without contributing to the condensation reaction (Fig. 5a, middle), whereas the nonanal adsorption itself is suppressed on ZnO/CuO nanowires (Fig. 5a, right). As a consequence of the suppressed aldol condensation reaction and reduced Zn^2+^ adsorption sites, the desorption amount of nonanal on the (Cu, Zn)O nanowire surface remains almost constant even when varying the CuO shell thickness (as seen in Fig. 4). Thus, the inactive surface Cu^2+^ site well explains the adsorption and the chemical transformation behaviors of nonanal on the ZnO/CuO nanowires and the (Cu, Zn)O nanowires.

In order to experimentally validate the above models, we analyze the adsorption states of molecules on nanowire surfaces *via* Fourier transform infrared spectroscopy (FTIR). Fig. 5b shows the FTIR spectra recorded at room temperature for nonanal-adsorbed ZnO nanowires, ZnO/CuO nanowires and (Cu, Zn)O nanowires (annealed at 500 °C) with various CuO shell thicknesses. The background signal from the nanowires is subtracted from each spectrum. The spectra show three peaks in the 1650–1710 cm^−1^ region corresponding to different *ν*(C=O) vibration modes of nonanal (*ν*_1_: 1706–1708 cm^−1^, *ν*_2_: ∼1700 cm^−1^, and *ν*_3_: 1658–1666 cm^−1^), where *ν*_1_ is seen in ZnO/CuO nanowires, *ν*_2_–*ν*_3_ are seen in ZnO nanowires and *ν*_1_–*ν*_3_ are seen in (Cu, Zn)O nanowires, respectively. Based on our DFT calculations, these peaks can be assigned to nonanal C=O coordinating at the (i) Cu^2+^ site (*ν*_1_), (ii) –OH (*ν*_2_) and (iii) Zn^2+^ site (*ν*_3_) (Fig. 5c, see details in ref. 31, ESI Fig. S12 and Table S3†). Two additional peaks around 1590 and 1550 cm^−1^ are observed for ZnO nanowires and 3 nm-thick (Cu,Zn)O nanowires, which are assigned to *(E)*-2-hepthyl-2-undecenal (*ν*_4_) and its oxidized species (*ν*_as_(COO)), respectively.^33^ These peaks are not seen when increasing the CuO shell thickness over 10 nm due to the suppressed aldol condensation reaction. Note that *ν*_3_ shows a lower shift upon introducing the CuO shell, indicating that the C=O bonding on the Zn^2+^ site becomes stronger. This is plausibly caused by the synergistic effect between Cu and Zn, which enhances the Lewis acidity of the Zn^2+^ site.^34^ However, the results in Fig. 4b indicate that such a synergistic effect does not govern the surface molecular behaviors. Next we investigate the thermal stability of each adsorption state in Fig. 5d–f. When increasing the temperature, the *ν*_1_ peak disappears at 100 °C, while *ν*_2_ and *ν*_3_ peaks disappear at 300 °C, showing that the C=O coordination of nonanal to the Cu^2+^ site is much weaker than that to the Zn^2+^ site. This result clearly identifies the validity of our model based on the inactive Cu^2+^ site. Note that *ν*_4_ and *ν*_as_(COO) peaks associated with the residual of *(E)*-2-hepthyl-2-undecenal and its oxidized species remain at 300 °C, anticipating their detrimental effects on catalytic and/or molecular sensing performances for low temperature operation. Thus our model for the surface molecular behaviors of nonanal on (Cu, Zn)O nanowires is experimentally confirmed by the surface spectroscopic analyses.

**Fig. 6:**
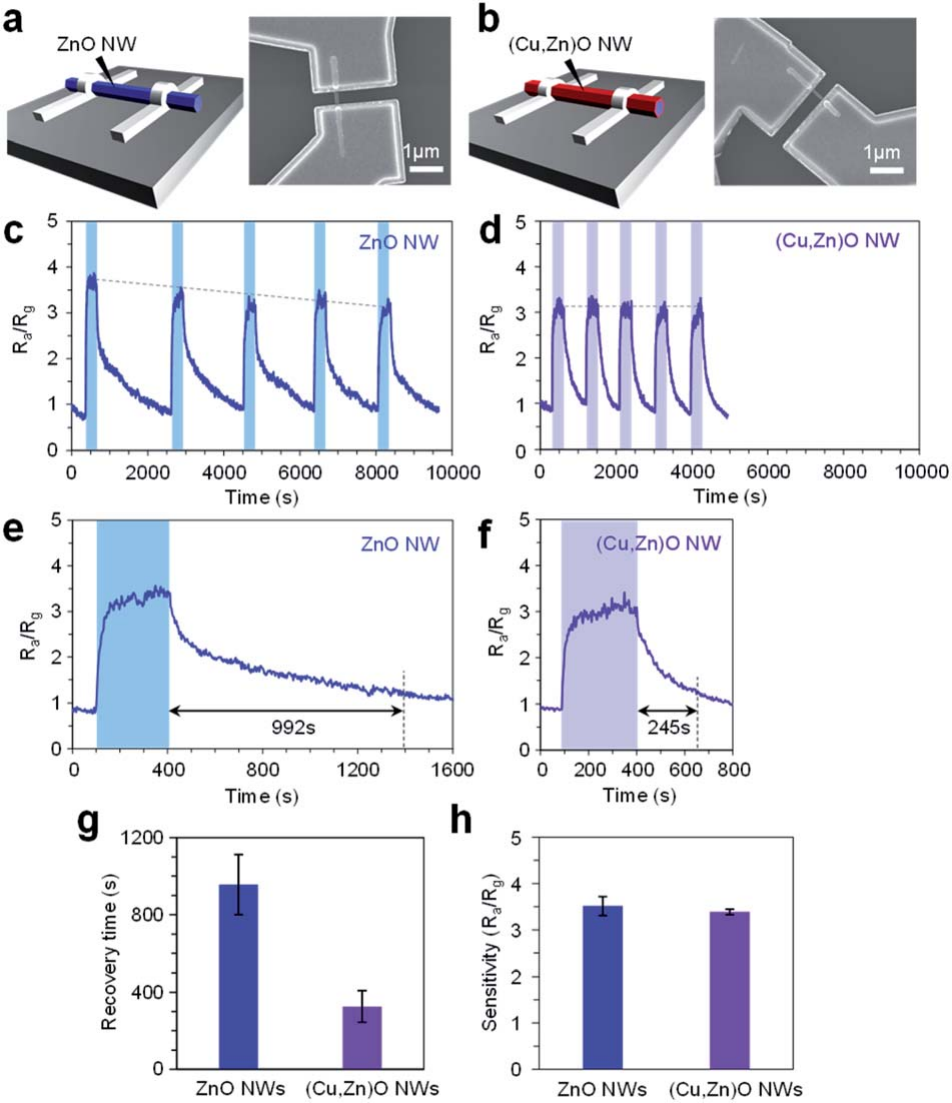
(a, b) Schematic illustrations and SEM images of single nanowire sensor devices for the (a) ZnO nanowire and (b) (Cu, Zn)O nanowire. The CuO thickness for the (Cu, Zn)O nanowire is 10 nm. (c, d) Successive five sensor responses and (e, f) typical single sensor response to 2.48 ppm nonanal for the (c, e) ZnO nanowire device and (d, f) (Cu, Zn)O nanowire device, respectively. All measurements were performed at 200 °C with a readout voltage of 1 V. (g) Recovery time and (h) sensitivity *R*_a_/*R*_g_ for the ZnO nanowire device and (Cu, Zn)O nanowire device. ‘NWs’ and ‘NW’ represent ‘nanowires’ and ‘nanowire’, respectively.

Finally we applied the mixed cation composition of the (Cu, Zn)O heterostructured nanowire surface for the electrical molecular sensing of nonanal. In this experiment, ZnO nanowires and 10 nm-thick (Cu, Zn)O nanowires annealed at 500 °C were examined in the form of a single nanowire device as shown in Fig. 6a and b, respectively. All devices exhibit linear current–voltage (I–V) characteristics, representing an Ohmic contact between the nanowires and the Pt electrodes (ESI Fig. S15†). The sensing measurements were conducted at 200 °C with a readout voltage of 1 V. The resistance ratio *R*_a_/*R*_g_, where *R*_a_ and *R*_g_ are resistance values in a N_2_ flow and 2.48 ppm nonanal flow respectively, was monitored as the sensor response. Fig. 6c and d show five successive sensor responses of the ZnO nanowire device and (Cu, Zn)O nanowire device, respectively, to nonanal. The sensor response of the ZnO nanowire device tends to deteriorate when increasing the number of sensing cycles. The result can be interpreted by the residual of the condensation product on the surface because the required temperature for fully desorbing *(E)*-2-hepthyl-2-undecenal (>300 °C, Fig. 4c and 5d) is higher than the operation temperature of the sensor. In contrast, the sensor response of the (Cu, Zn)O nanowire device is almost the same even when increasing the number of sensing cycles, reflecting a suppressed condensation reaction. The effect of suppressed molecular condensation is also found in the recovery response, in which the recovery time in the ZnO nanowire device (992 s) becomes much shorter in the (Cu, Zn)O nanowire device (245 s) (Fig. 6e–g). As such, our results show that the durability and the recovery time of the nanowire sensor can be successfully improved by suppressing the aldol condensation reaction. It is worth describing that all these improvements in the sensor performance of the (Cu, Zn)O nanowire device were demonstrated by maintaining the sensitivity (Fig. 6h). Since the aldol condensation reaction is suppressed on (Cu, Zn)O nanowires, the observed sensor response is solely associated with the adsorption of nonanal. This is well consistent with the fact that the aldol condensation reaction does not involve charge transfer between the molecule and sensor surface. Since the amount of adsorbed nonanal is maintained as a consequence of a controlled number of Zn^2+^ adsorption sites and suppressed molecular condensation (Fig. 4), the preserved sensitivity of the (Cu, Zn)O nanowire sensor is reasonable. Thus the continuous modulation of surface cation composition by a post-growth thermal treatment is a rational way to understand and tailor the surface molecular behaviors. Since this approach can be extended to diverse metal oxides by varying their material combination, it paves a novel way to design a nanoscale metal oxide surface for enhancing the performances of various applications such as molecular sensors and catalysts.

## Conclusion

In conclusion, we demonstrated a gradual modulation of the surface cation composition of ZnO/(Cu_1−*x*_Zn_*x*_)O heterostructured nanowires by cation diffusion and investigated the effect of the surface cation composition on the adsorption and chemical transformation behaviors of nonanal. The gradual modulation of the surface Cu/Zn ratio was successfully achieved by preserving the macroscopic morphology. We found that surface exposed Cu^2+^ significantly suppresses the adsorption of nonanal and the increase of the surface Cu/Zn ratio systematically suppresses the molecular condensation reaction, although the Lewis acidity of Cu^2+^ is strong enough and comparable to that of Zn^2+^. Surface spectroscopic analysis and theoretical simulations revealed that the observed adsorption behaviors of nonanal can be interpreted in terms of the coordination-saturated in-plane square geometry of surface Cu^2+^ sites. The presence of inactive surface Cu^2+^ sites well explains the mechanism of suppressed surface aldol condensation reactions by preventing the neighboring of Zn^2+^ sites. As a consequence of controlled surface cation composition in ZnO/(Cu_1−*x*_Zn_*x*_)O nanowires, we successfully demonstrated significant improvements in the durability and the recovery time of nonanal sensing in nanowire sensors while maintaining the sensitivity. The results highlighted the critical importance to continuously modulate the surface cation composition for understanding and tailoring the surface molecular behaviors on the nanoscale metal oxide surface.

## Experimental

### Preparation of nanowire samples

ZnO nanowires were grown on a 100 nm thick SiO_2_ coated Si (100) substrate by hydrothermal synthesis.^36–41^ A 5 nm thick Ti buffer layer and a 100 nm thick ZnO film were sequentially deposited on the substrate by radio frequency (RF) sputtering at a RF power of 50 W and an Ar pressure of 0.3 Pa. The substrate was immersed into a 150 ml aqueous solution containing 25 mM zinc nitrate hexahydrate (Zn(NO_3_)_2_·6H_2_O, Wako 99.0%) and 25 mM hexamethylenetetramine (HMTA, (CH_2_)_6_N_4_ Wako 99.0%) with the top side down. The ZnO nanowires were grown by keeping the solution at 95 °C for 15 h in an oven. After the growth, the samples were taken out from the solution and rinsed with water. CuO/ZnO nanowires were fabricated by depositing the CuO shell on the as-grown ZnO nanowires by means of pulsed laser deposition (PLD) with a repetition rate of 10 Hz at room temperature in a 1 Pa oxygen atmosphere. A CuO(ii) tablet (99.9% pure) was utilized as a target for the PLD measurement. The thickness of the CuO shell was controlled by varying the deposition time. For the ZnO/CuO nanowires, an oxygen plasma treatment was performed at 150 W in a 1 Torr oxygen atmosphere after depositing the CuO layer. The ZnO nanowires and the (Cu, Zn)O nanowires were prepared by a post-growth thermal treatment at various annealing temperatures in the range of 200–1000 °C for 3 h in air.

### Characterization of the nanowires

The morphology, crystal structure and composition of the samples were characterized by scanning electron microscopy (SEM, JEOL JSM-7610F) at an accelerating voltage of 15 kV, X-ray diffraction (XRD, Rigaku TTR-II) with a Cu Kα radiation source at 50 kV and 300 mA, transmission electron microscopy (TEM, Thermo Fisher Scientific Titan Cubed 60-300 G2) coupled with energy dispersive X-ray spectroscopy (EDS) at an accelerating voltage of 300 kV and X-ray photoelectron spectroscopy (XPS, Kratos AXIS-ULTRA) with an Al Kα radiation source (12 kV, 5 mA, monochromator). For XPS, the electron escape depth is *ca.* 1–2 nm and the binding energies were corrected using the C 1s peak. The CuO shell thickness was estimated from the Cu/Zn ratio obtained by EDS analysis and the total diameter of the nanowires. A Fourier transform infrared spectroscope (FTIR, Thermo Fisher Scientific Nicolet iS50) equipped with a MCT (HgCdTe) detector was used to evaluate the adsorbed molecules on the nanowires. For monitoring the temperature dependent variations of the adsorbed state of molecules, the samples were heated at a given temperature for 5 min and immediately cooled down to room temperature in air. All FTIR measurements were performed at room temperature.

### Analysis of desorbed molecules

For the desorbed molecule analysis, firstly nonanal was adsorbed onto the nanowire surface. The nanowire samples were cut into a piece of 2 mm × 20 mm. 2 μl liquid nonanal was placed at the bottom of a 25 ml vial bottle, followed by closing the bottle and keeping for 30 min to vaporize nonanal. The suspended samples were inserted in the bottle filled with nonanal vapor, kept for 30 s and transferred to a gas chromatography mass spectrometry (GCMS, SHIMADZU GCMS-QP2020 Ultra) system. The desorbed compounds were analyzed by using the GCMS equipped with an inlet temperature control unit (OPTIC4) using a Supelco SLB-IL60 capillary column. During the measurement, the inlet temperature was kept at 400 °C. The desorption temperature of the compounds was evaluated *via* temperature-programmed desorption mass spectrometry (TPDMS) measurements. A capillary column (length: 1 m; inner diameter: 0.1 mm) without stationary phases was utilized to connect between the inlet and mass spectrometer units. The inlet temperature was controlled from 35 °C to 500 °C by using a multi-shot pyrolyzer (FRONTIER LAB, EGA/PY-3030D) with a heating rate of 0.5 °C s^−1^. The variations in total ion currents (TICs) were monitored during elevating the inlet temperature.

### Computational details

We computed the vibrational frequencies of nonanal on ZnO (101̄0) and CuO (111) surfaces using density functional theory (DFT) to assign the C=O stretching bands. Simplified cluster models were employed to consider the adsorbed ZnO (101̄0) and CuO (111) surface approximately. These models were extracted from a Wurtzite ZnO crystal structure with *a* = *b* = 3.25 Å and *c* = 5.2 Å, or a monoclinic CuO crystal structure with *a* = *b* = 2.93 Å and *c* = 5.15 Å. In order to mimic the above-mentioned surfaces, unsaturated metal–oxygen bonds are capped and neutralized with protons placed on the line segments between oxygens and points where neighbouring Zn^2+^ or Cu^2+^ originally exist. The O–H bond lengths are set as 0.957 Å. The DFT calculations were carried out using the Gaussian 16 program suite Revision A03 with the B3LYP hybrid functional.^42^ The basis set employed was def2-SVP. All the cluster atoms were kept fixed during geometry optimization. The obtained harmonic vibrational frequencies were shifted using a scale factor of 0.93, which was determined as the ratio of calculated and experimental C=O vibrational frequencies of acetaldehyde,^43^ to incorporate anharmonic effects effectively. The adsorption energies of C=O − M^+^ are calculated as the differences of the total energy (*E*_total_) of the optimized adsorbed structure (C=O − M^+^) and desorbed structure (C=O + M^+^) (ESI Fig. S11†).

### Preparation of single nanowire sensors and molecular sensing measurements

The nanowire suspension was prepared by sonicating the fabricated nanowire samples in 2-propanol. The nanowire was transferred by adding dropwise the suspension on a 100 nm thick SiO_2_ coated Si (100) substrate with pre-patterned contact electrodes. Electron beam (EB) lithography (JEOL, JSM-7001F and SANYU electron, NANO PRINTER SPG-724) was performed at an accelerating voltage of 30 kV to make the electrode patterns for bridging a single nanowire between them using ZEP520A-7 (ZEON) as a resist. For the electrical contact, Pt was deposited by RF sputtering with a 300 nm thickness. After a lift-off process in *N*,*N*-dimethylformamide (DMF) and a rinse process in acetone, a single nanowire sensor device was obtained. The gap size of bridged nanowires was designed to be 500 nm and the nanowires with a *ca.* 100 nm diameter were chosen for all fabricated devices to eliminate the variation of electrical properties caused by the device geometry. Molecular sensing measurements were conducted using a customized probe station (JANIS, ST-500) connected with a gas flow system and a semiconductor parameter analyzer (Tektronix, Keithley 4200-SCS). The variations of the electrical signal under flowing N_2_ or 2.48 ppm nonanal were monitored by applying a voltage of 1 V to the electrodes. All measurements were performed at 200 °C. The sensor response was defined as *R*_a_/*R*_g_, where *R*_a_ and *R*_g_ are the sensor resistance when exposed to N_2_ and nonanal, respectively. The sensor recovery time was defined as the time for reaching 90% of *R*_a_ after stopping the nonanal flow. We evaluated several nanowire sensor devices for the reliability of results shown in this study.

## Conflicts of interest

There are no conflicts to declare.

## Supplementary Material

SC-012-D1SC00729G-s001
