# Supplementary material for: The impact of surface Cu2+ of ZnO/(Cu1−xZnx)O heterostructured nanowires on the adsorption and chemical transformation of carbonyl compounds
Source: Chem Sci. 2021 Mar 8;12(14):5073–81. doi: 10.1039/d1sc00729g (PMC8179607; doi:10.1039/d1sc00729g)
Supplement: SC-012-D1SC00729G-s001 [file SC-012-D1SC00729G-s001.pdf]

Electronic Supplementary Material (ESI) for Chemical Science.
This journal is © The Royal Society of Chemistry 2021

Electronic supplementary information

The impact of surface Cu^{2+} of $ZnO/(Cu_{1-x}Zn_x)O$ heterostructured nanowires on the adsorption and chemical transformation of carbonyl compounds

Jiangyang Liu[1], Kazuki Nagashima[1,2]*, Yuki Nagamatsu[3], Takuro Hosomi[1,2], Hikaru Saito[3], Chen Wang[3], Wataru Mizukami[2,4,5], Guozhu Zhang[1], Benjarong Samransuksamer[1], Tsunaki Takahashi[1,2], Masaki Kanai[3], Takao Yasui[2,6], Yoshinobu Baba[6] and Takeshi Yanagida[1,3]*

[1]*Department of Applied Chemistry, Graduate School of Engineering, The University of Tokyo, 7-3-1 Hongo, Bunkyo-ku, Tokyo 113-8656, Japan*

[2]*Japan Science and Technology Agency (JST), PRESTO, 4-1-8 Honcho, Kawaguchi, Saitama 332-0012, Japan*

[3]*Institute for Materials Chemistry and Engineering, Kyushu University, 6-1 Kasuga-Koen, Kasuga, Fukuoka 816-8580, Japan*

[4]*Center for Quantum Information and Quantum Biology, Institute for Open and Transdisciplinary Research Initiatives, Osaka University, 1-3 Machikaneyama, Toyonaka, Osaka 560-8531, Japan*

[5]*Graduate School of Engineering Science, Osaka University, 1-3 Machikaneyama, Toyonaka, Osaka 560-8531, Japan*

[6]*Department of Biomolecular Engieering, Graduate School of Engineering, Nagoya University, Furo-cho, Chikusa-ku, Nagoya 464-8603, Japan*

Corresponding author's e-mail: kazu-n@g.ecc.u-tokyo.ac.jp (K.N.),

yanagida@g.ecc.u-tokyo.ac.jp (T.Y.)

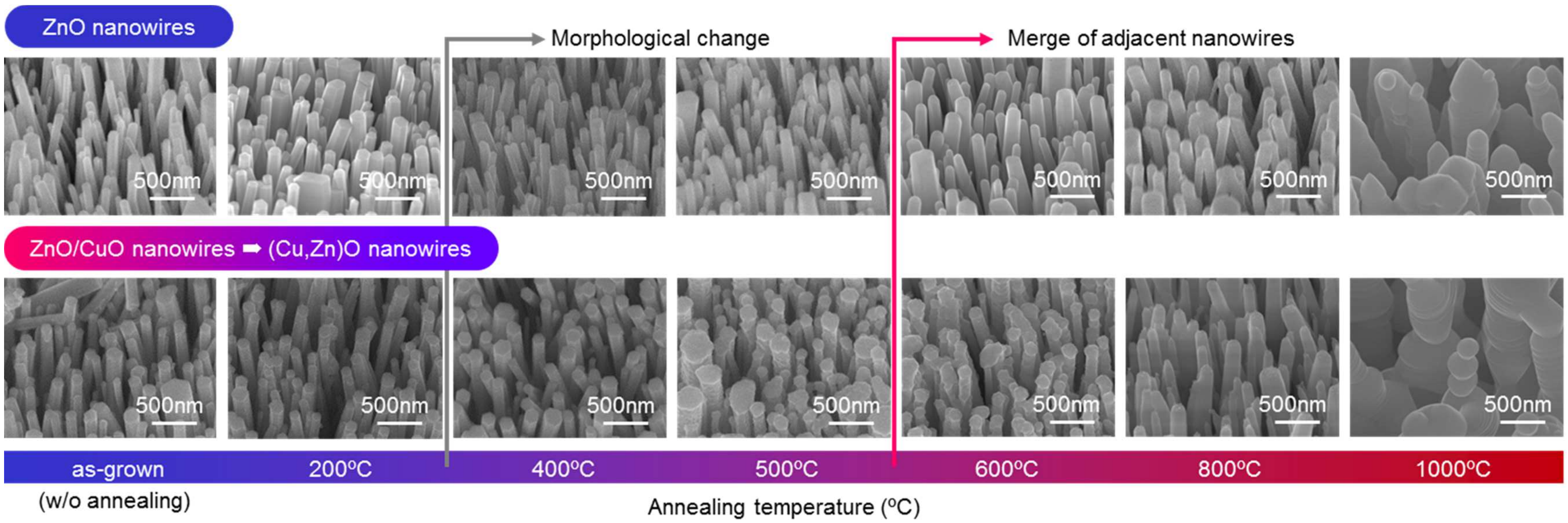

Fig. S1. Series of SEM images for ZnO nanowires and (Cu,Zn)O nanowires when varying annealing temperature. The CuO shell thickness is ca. 10 nm. The morphological change is observable at above 400 ºC while a merge of adjacent nanowires is seen at above 600 ºC.

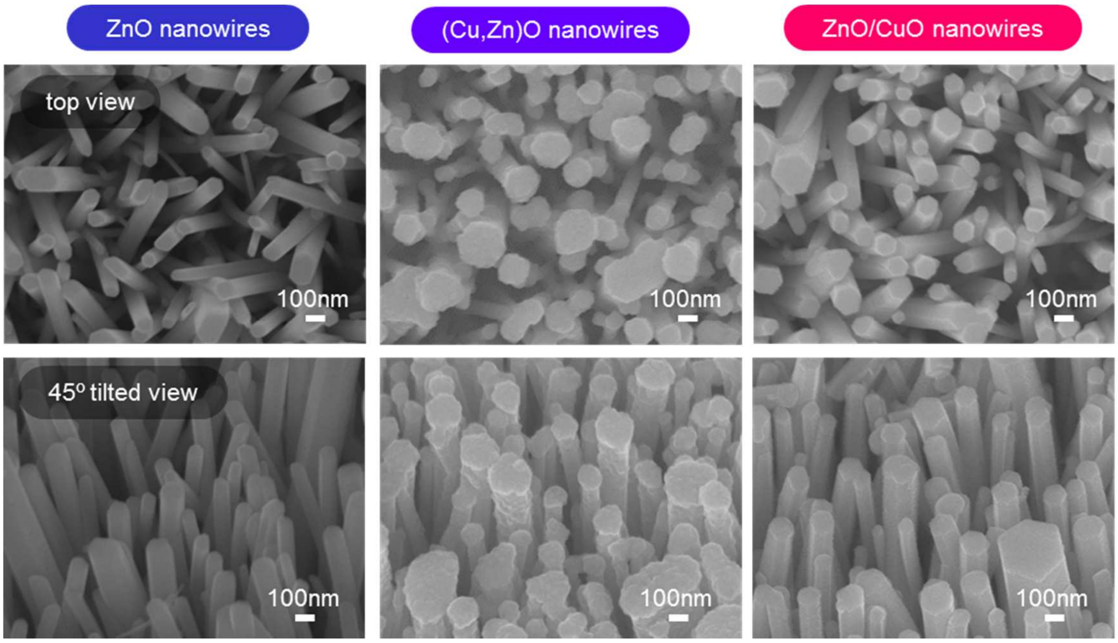

Fig. S2. Top view and 45° tilted SEM images of ZnO nanowires, (Cu,Zn)O nanowires and ZnO/CuO nanowires. The CuO shell thickness is ca. 10 nm. The annealing temperature of ZnO nanowires and (Cu,Zn)O nanowires is 500 ºC

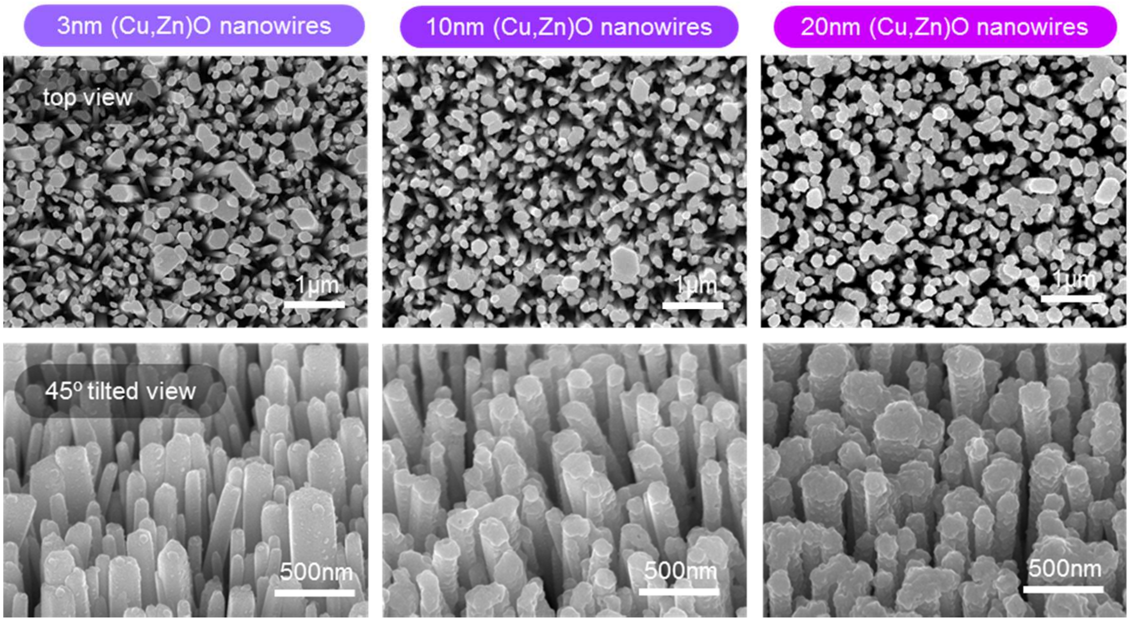

Fig. S3. Top view and 45° tilted SEM images of (Cu,Zn)O nanowires with various CuO shell thickness (3 nm, 10 nm, 20 nm). The annealing temperature is 500 ºC

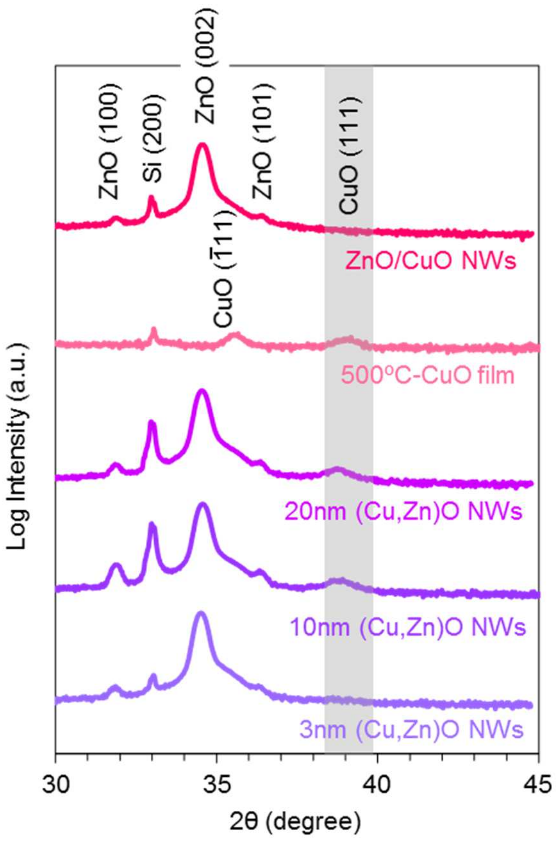

Fig. S4. XRD patterns of (Cu,Zn)O nanowires, CuO film and ZnO/CuO nanowires. For (Cu,Zn)O nanowires, the CuO thickness is varied in the range of 3-20 nm. The annealing temperature for (Cu,Zn)O nanowires is 500 ºC. CuO film with 200 nm thickness is annealed at 500 ºC for 1 h in air prior to measurement.

Sample	2θ (degree)	*d* value (Å)
ZnO/CuO NWs	-	-
CuO film	39.167	2.299
20nm (Cu,Zn)O NWs	38.807	2.319
10nm (Cu,Zn)O NWs	38.757	2.322
3nm (Cu,Zn)O NWs	-	-

Table S1. 2θ values and *d* values of CuO (111) peaks.

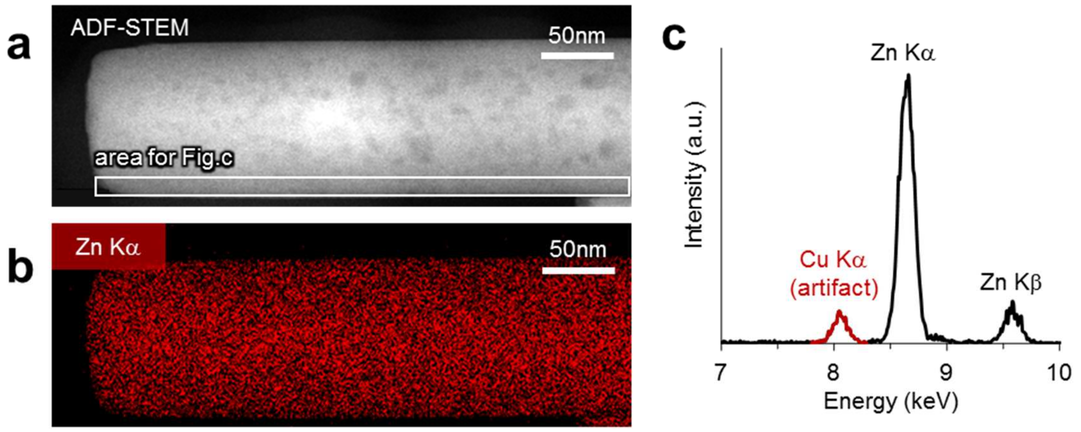

Fig. S5. (a) ADF-STEM image, (b) Zn map and (c) EDS spectrum of a ZnO nanowire. The EDS spectrum in (c) was extracted from the area projected by the white rectangle in (a). Since the artifact Cu peak in (c) originates from the sample holder, it was subtracted for the EDS spectra in Fig.1 (m-o) and the calculation of Zn concentration in CuO shell.

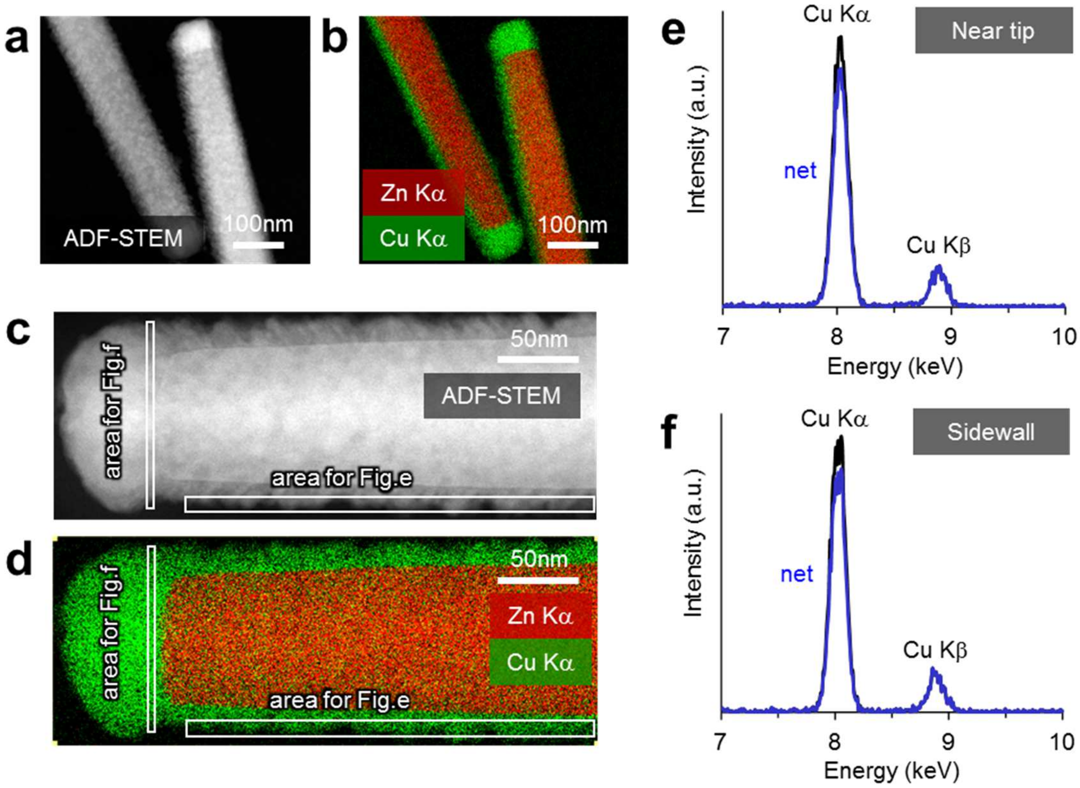

Fig. S6. (a,c) ADF-STEM images, (b,d) EDS elemental maps and (e,f) EDS spectra of ZnO/CuO nanowires. The EDS spectra in (e) (near tip) and (f) (sidewall) were extracted from the areas projected by the white rectangles in (c) and (d). Net spectra (blue curves in (e) and (f)) were calculated by subtracting the artifact peak (red peak in Fig. S6 (c)) from the raw data (black curves). No significant Zn peak was observed in (e) and (f).

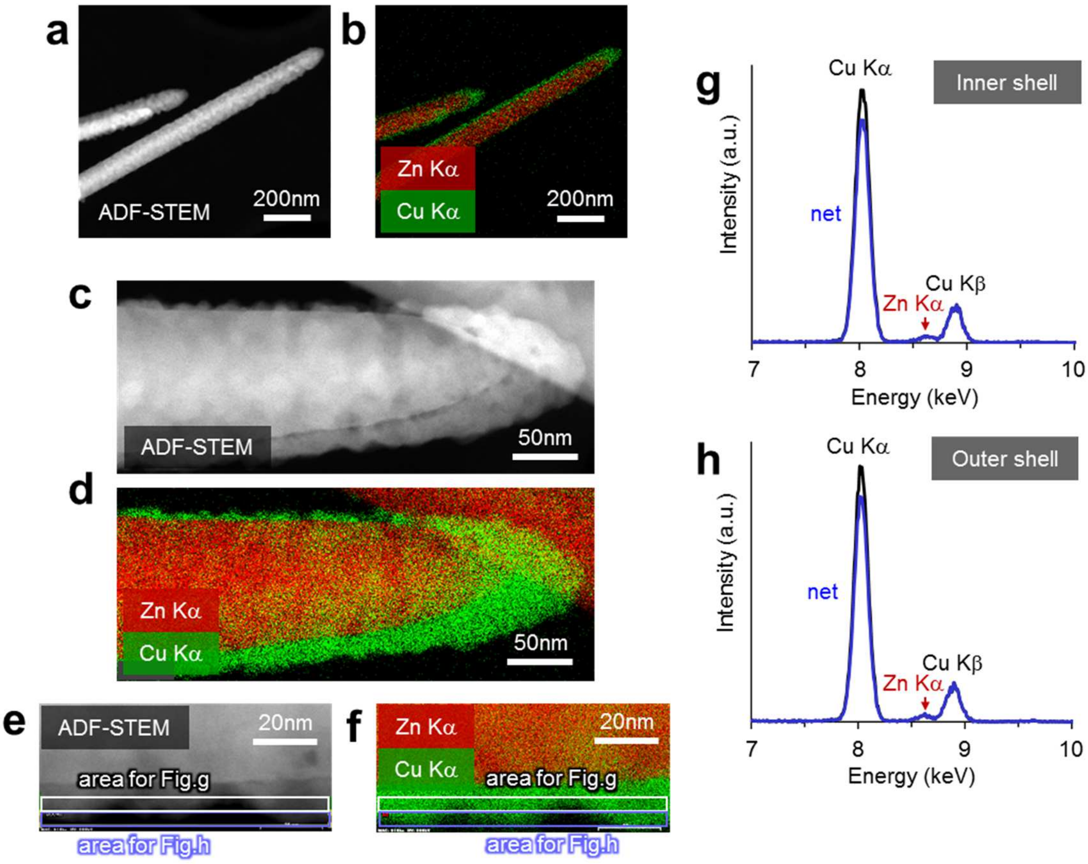

Fig. S7. (a,c,e) ADF-STEM images, (b,d,f) EDS elemental maps and (g,h) EDS spectra of (Cu,Zn)O nanowires annealed at 400 ℃. The EDS spectra in (g) (inner shell) and (h) (outer shell) were extracted from the areas projected by the white and blue rectangles in (e) and (f). Net spectra (blue curves in (g) and (h)) were calculated by subtracting the artifact peak (red peak in Fig. S6(c)) from the raw data (black curves). The presence of Zn Kα in the CuO layer indicates the occurrence of Zn diffusion during the thermal annealing. The Zn concentration calculated using net spectra were 4.5 % for both (g) and (h).

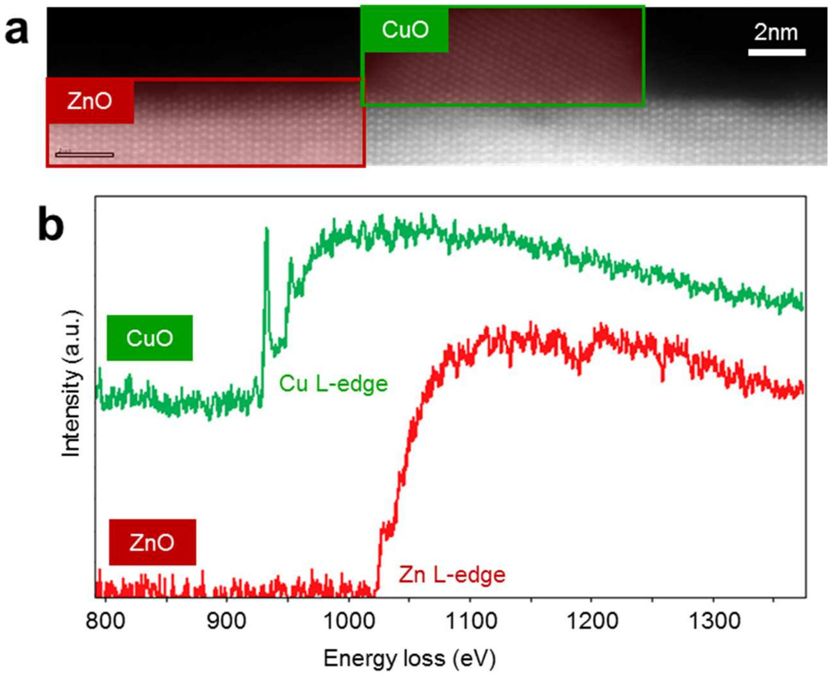

Fig. S8. (a) ADF-STEM image and (b) EELS spectra of (Cu,Zn)O nanowire surface. The EELS spectra were extracted from the areas projected by the green (CuO shell) and red (ZnO nanowire) rectangles in (a), respectively. No significant Cu peak was observed at ZnO nanowire surface.

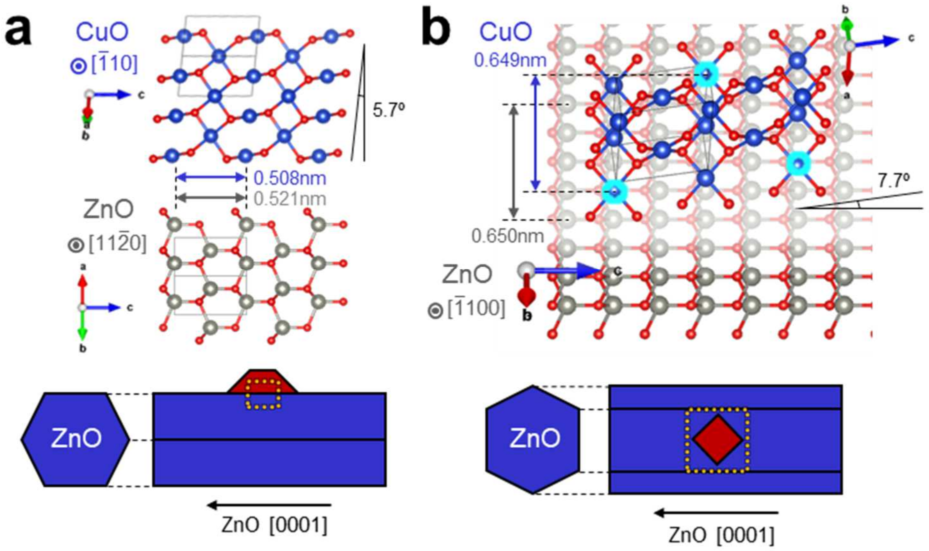

Fig. S9. Crystal structures of ZnO and CuO and their orientations at interface, which are projected along (a) ZnO $[11\bar{2}0]$ direction (left) and (b) ZnO $[\bar{1}100]$ (right), respectively. The schematic illustrations of nanowires corresponding to each crystal structure are also shown (lower images).

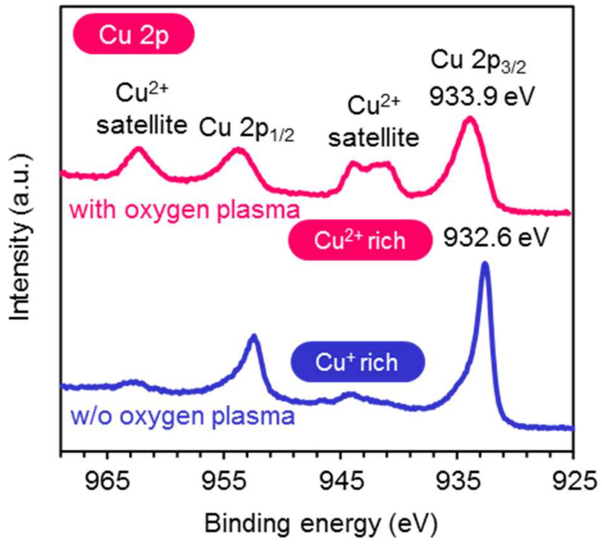

Fig. S10. Cu 2p XPS spectra of ZnO/CuO nanowires before and after performing oxygen plasma treatment.

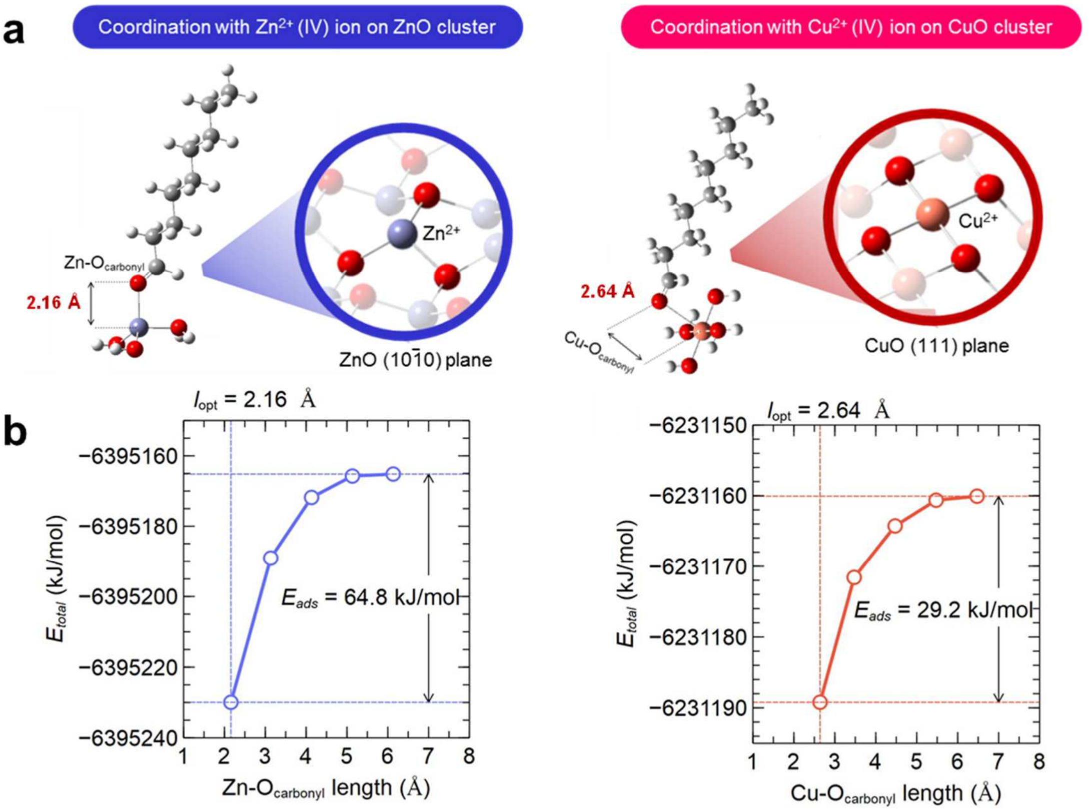

Fig. S11. (a) Computed structures and calculated bond lengths of nonanal C=O—metal ions (Zn^{2+} and Cu^{2+}) by using simplified models of ZnO $(10\bar{1}0)$ plane and CuO (111) plane. Unsaturated metal-oxygen bonds are capped and neutralized with protons. Relative positions of oxygens and hydrogens are fixed during the geometrical optimizations (Table S3). (b) Calculated nonanal adsorption energies (E_{ads}) on ZnO $(10\bar{1}0)$ plane and CuO (111) planes. The desorption structures were defined as the point where the total energies (E_{total}) are saturated against metal-$O_{carbonyl}$ length increase.

model cluster	bond length (Å)	adsorption energy (kJ/mol)
ZnO: $(10\bar{1}0)$ plane	2.16	64.8
CuO: (111) plane	2.64	29.2

Table S2. Simulated bond lengths and adsorption energies of nonanal on simplified structures of ZnO $(10\bar{1}0)$ or CuO (111).

To examine the effect of coordination degree of surface metal ions, we conducted the density functional theory (DFT) calculations for the nonanal molecules adsorbed on CuO (111) plane and ZnO $(10\bar{1}0)$ plane. The estimated length of C=O—M^+ bonding is much longer on CuO (2.64 Å) than that on ZnO (2.16 Å), and the adsorption energy E_{ads} of nonanal—ZnO is much higher than that of nonanal—CuO (Table S2). These results suggest the weaker adsorption of nonanal on CuO (111) plane.

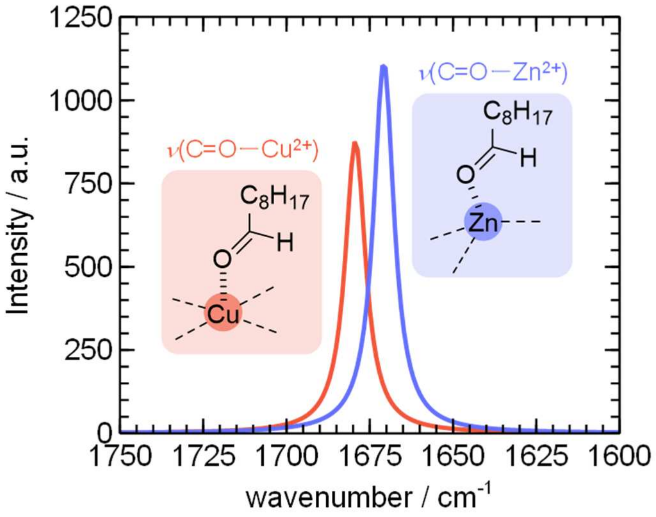

Fig. S12. Simulated infrared spectra for adsorbed nonanal on ZnO $(10\bar{1}0)$ (blue line) and CuO (111) (orange line), respectively. The molecular geometries used to calculate these vibrational frequency calculations are summarized in the Table S3.

(a) C=O—ZnO $(10\bar{1}0)$ plane

element	x	y	z	optimization
O	-5.79872	-1.08669	-0.18803	fix
O	-3.83707	0.59073	1.808985	fix
O	-4.74613	1.912112	-1.03259	fix
Zn	-4.26016	0.197426	-0.11937	fix
H	-6.00003	-1.27416	-1.10464	fix
H	-4.94745	1.724619	-1.9492	fix
H	-3.94024	1.53023	1.959138	fix
H	-4.44279	0.098033	2.362343	fix
O	-2.28036	-0.5855	-0.47647	move
C	-1.3335	-0.67167	0.285093	move
H	-1.44137	-0.31487	1.336833	move
C	-0.00061	-1.2285	-0.10096	move
C	1.152987	-0.2502	0.190251	move
H	-0.03436	-1.5205	-1.1618	move
H	0.146162	-2.14775	0.499381	move
C	2.529593	-0.84111	-0.12918	move
H	1.002033	0.672469	-0.39708	move
H	1.120015	0.053197	1.252489	move
C	3.686052	0.125957	0.140092	move
H	2.675214	-1.76387	0.46236	move
H	2.552779	-1.15579	-1.18829	move
H	3.535397	1.047789	-0.45149	move
H	3.658881	0.44195	1.199547	move
C	5.064809	-0.46034	-0.17826	move
C	6.223795	0.504283	0.088936	move
H	5.090008	-0.77684	-1.23755	move
H	5.214064	-1.38309	0.412858	move
C	7.603436	-0.08058	-0.2295	move
H	6.074191	1.427194	-0.50212	move
H	6.198334	0.821294	1.148452	move
C	8.755547	0.88834	0.03977	move
H	7.751094	-1.00314	0.361221	move
H	7.627057	-0.39713	-1.28826	move
H	9.730481	0.43496	-0.20016	move
H	8.65784	1.805705	-0.56479	move
H	8.783078	1.194603	1.099079	move

(b) C=O—CuO (111) plane

element	x	y	z	optimization
Cu	3.571491	0.641622	-0.16127	fix
O	5.487489	0.257725	-0.07168	fix
O	3.298999	0.094719	-2.01269	fix
O	3.844759	1.184851	1.688043	fix
O	1.655617	1.022826	-0.25274	fix
H	3.493482	-0.83175	-2.15212	fix
H	5.682069	-0.66887	-0.2106	fix
H	3.650739	2.112536	1.828079	fix
H	1.461295	1.949597	-0.11424	fix
H	2.361556	0.282253	-2.05761	fix
H	4.782525	0.9995	1.733833	fix
O	2.518001	-1.77314	0.004296	move
C	1.526319	-1.51431	0.656965	move
H	1.617134	-0.97185	1.628398	move
C	0.127696	-1.91718	0.275246	move
C	-0.911	-0.80928	0.496042	move
H	-0.12652	-2.79698	0.900726	move
H	0.146167	-2.26368	-0.77109	move
C	-2.32903	-1.22018	0.093475	move
H	-0.90252	-0.5125	1.561046	move
H	-0.5798	0.07967	-0.06434	move
C	-3.36726	-0.1132	0.299187	move
H	-2.3321	-1.52796	-0.96886	move
H	-2.63325	-2.11784	0.664494	move
H	-3.3687	0.192122	1.362285	move
H	-3.0565	0.783751	-0.26787	move
C	-4.78755	-0.50892	-0.11499	move
C	-5.82596	0.597669	0.090828	move
H	-5.0957	-1.40773	0.451348	move
H	-4.78542	-0.81333	-1.17836	move
C	-7.24663	0.20295	-0.32475	move
H	-5.82935	0.901976	1.154453	move
H	-5.51789	1.497005	-0.47496	move
C	-8.27731	1.313375	-0.11588	move
H	-7.24202	-0.1009	-1.38758	move
H	-7.55309	-0.69602	0.240858	move
H	-9.28576	0.994775	-0.425	move
H	-8.33401	1.613817	0.944012	move
H	-8.02013	2.214038	-0.69855	move

Table S3. Optimized molecular geometries of nonanal on (a) ZnO $(10\bar{1}0)$ and (b) CuO (111) used for vibrational frequency calculations. The “optimization” column describes whether each atom is fixed or moved during the geometrical optimization.

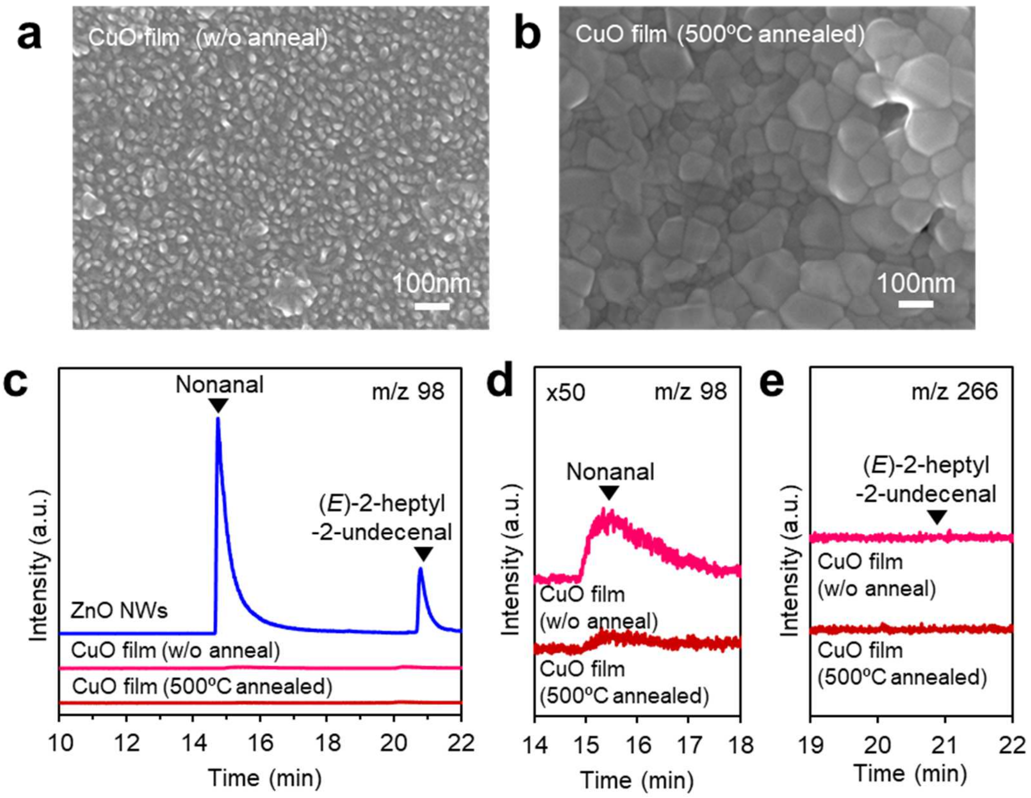

Fig. S13 SEM images of (a) CuO film (oxygen plasma is applied, w/o annealing) and (b) CuO film annealed at 500 ºC. (c-d) GCMS spectra for desorption compounds on CuO films; (c) overview, (d) the magnified data around nonanal peak (m/z 98) and (e) the magnified data around (*E*)-2-heptyl-2-undecenal peak (m/z 266), respectively. As a control, the data of ZnO nanowires is also shown. 'NWs' abbreviates 'nanowires'.

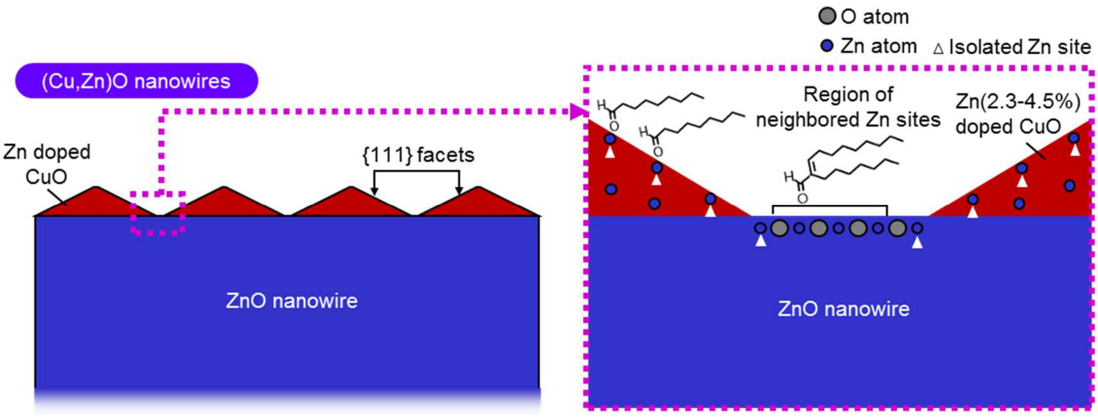

Fig. S14. A model for the molecular behaviors of nonanal on (Cu,Zn)O nanowire surface. The neighboring of surface Zn sites is prevented on Zn doped CuO surface and at the periphery of ZnO/CuO interface.

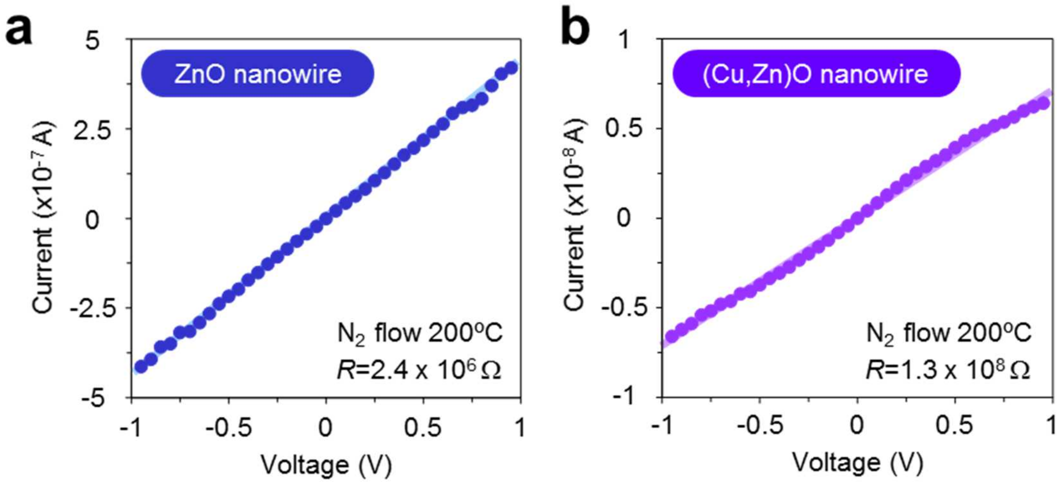

Fig. S15. Current-voltage (*I*-*V*) curves of single nanowire devices composed of (a) ZnO nanowire and (b) (Cu,Zn)O nanowire annealed at 500 ºC (CuO shell thickness: 10 nm), respectively. Measurements are performed at 200 ºC under N_2 flow.
